# Supplementary material for: In vitro lung epithelial cell model reveals novel roles for Pseudomonas aeruginosa siderophores
Source: Microbiol Spectr. 2024 Feb 5;12(3):e03693-23. doi: 10.1128/spectrum.03693-23 (PMC10913452; doi:10.1128/spectrum.03693-23)
Supplement: Figures S1-S5 — Supplemental figures S1-S5 with their legends. [file spectrum.03693-23-s0001.pdf]

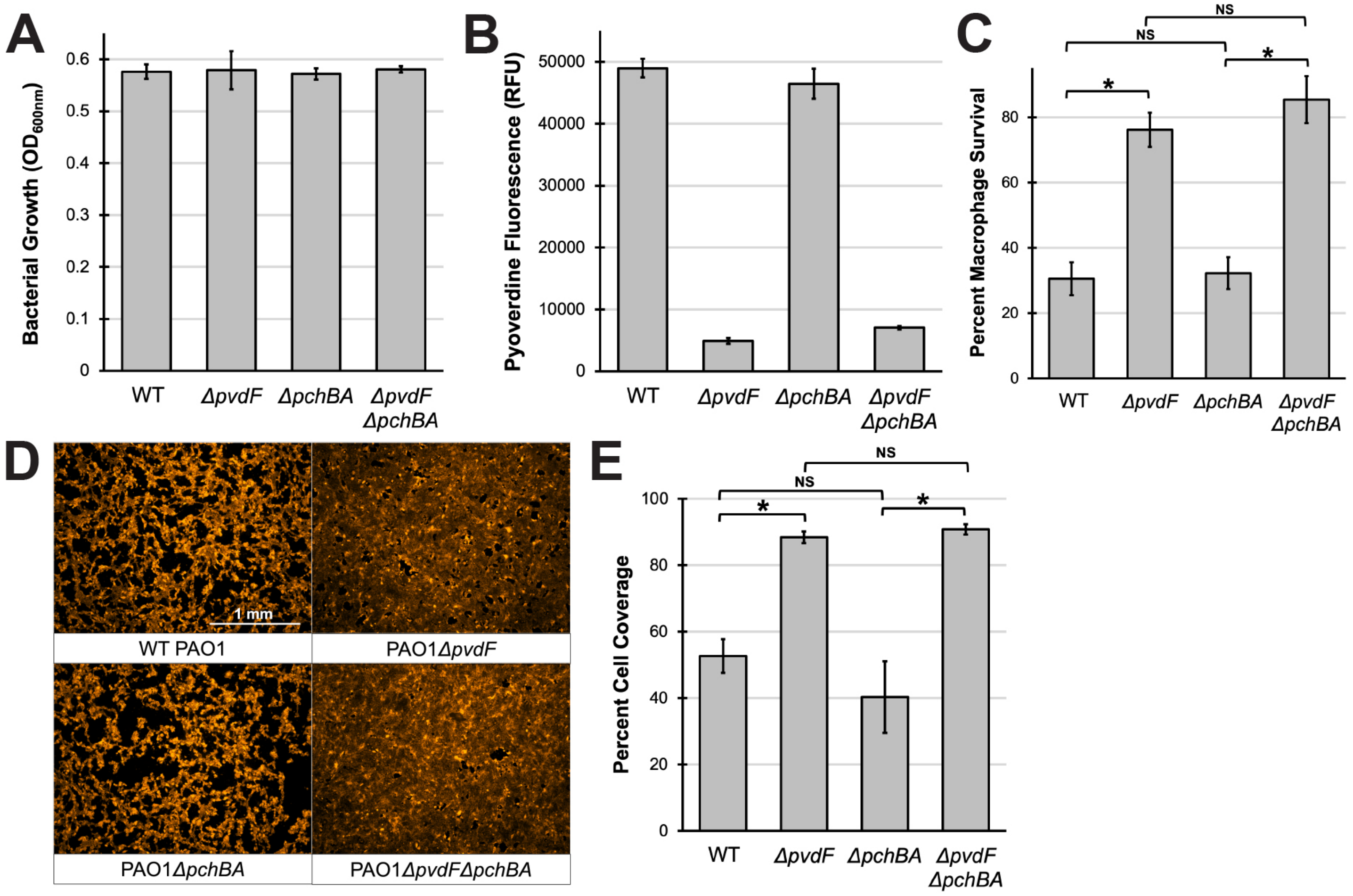

Fig. S1. Disruption of pyochelin biosynthesis does not mitigate epithelial damage. (A, B) Bacterial growth **(A)** or pyoverdine production **(B)** of *P. aeruginosa* PAO1 siderophore biosynthetic mutants (PAO1*ΔpvdF* – pyoverdine; PAO1*ΔpchBA* – pyochelin; PAO1*ΔpvdFΔpchBA* – pyoverdine and pyochelin) after 18 h growth in serum-free EMEM. **(C)** MH-S murine alveolar macrophage viability after 2 h exposure to conditioned medium from PAO1 siderophore mutants grown in EMEM. Cell viability was measured using a resazurin-based assay. **(D)** Fluorescent micrographs of 16HBE cells after 30 min exposure to conditioned medium from PAO1 siderophore mutants. Cells were prelabeled with CellMask Orange plasma membrane stain. **(E)** Quantification of percentage micrograph area covered by fluorescent cells. All error bars represent SEM from four biological replicates. * corresponds to $p < 0.01$ and NS corresponds to $p > 0.05$ based on one-way ANOVA with Tukey's multiple comparisons test.

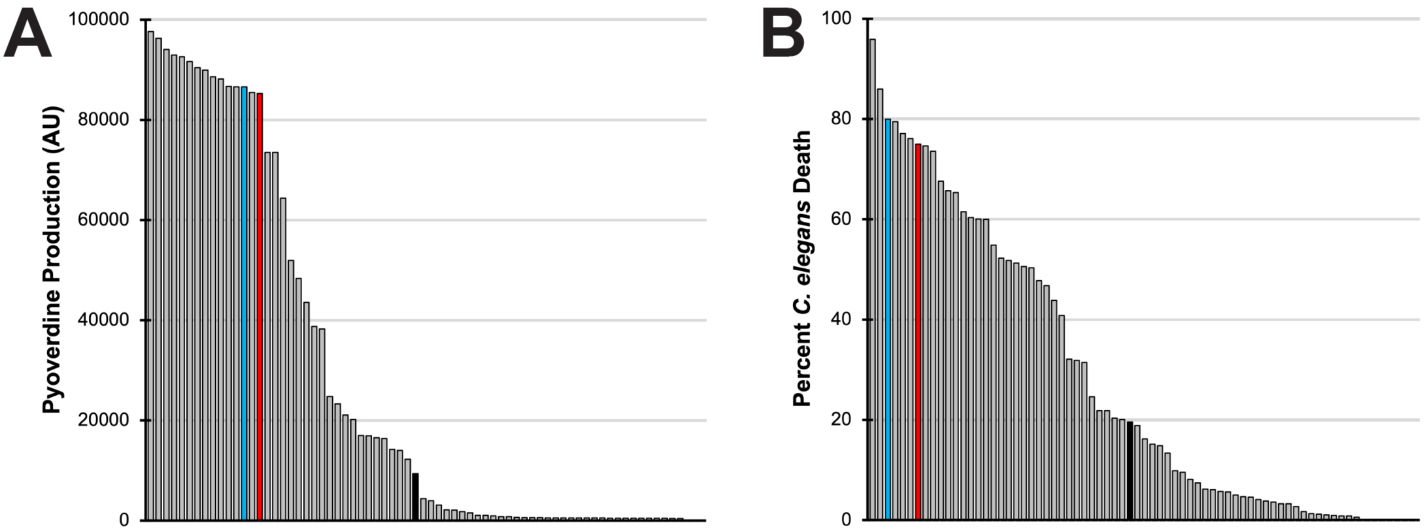

Fig. S2. Pyoverdine production and *C. elegans* killing by *P. aeruginosa* clinical isolates from pediatric cystic fibrosis patients. (A, B) Pyoverdine production **(A)** or killing of *C. elegans* **(B)** by multidrug-resistant *P. aeruginosa* strains isolated from pediatric cystic fibrosis patients. Black bar represents the median pyoverdine production or *C. elegans death*. Red bar represents PA2-72, blue bar represents PA2-61. Survey data was adapted from (22).

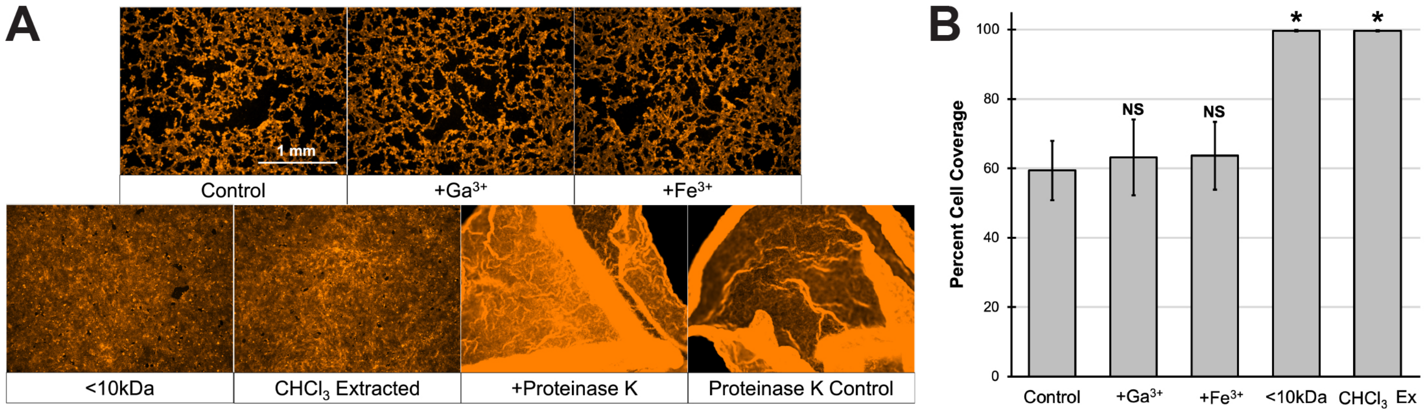

Fig. S3. Lipid extraction abrogates epithelial damage by pyoverdine-rich conditioned medium. (A) Fluorescent micrographs of 16HBE cells after 30 min exposure to conditioned medium from WT PAO1. Conditioned medium was pretreated with 200 µM $Ga(NO_3)_3$, 200 µM $FeCl_3$, or 100 µg/mL proteinase K for 24 h, had macromolecules depleted via a 10 kDa centrifugal filter, or had lipids depleted by chloroform ($CHCl_3$) extraction. Cells were prelabeled with CellMask Orange plasma membrane stain. **(B)** Quantification of percentage micrograph area covered by fluorescent cells. Error bars represent SEM from three biological replicates. * corresponds to $p < 0.01$ and NS corresponds to $p > 0.05$ based on one-way ANOVA with Dunnett's multiple comparisons test.

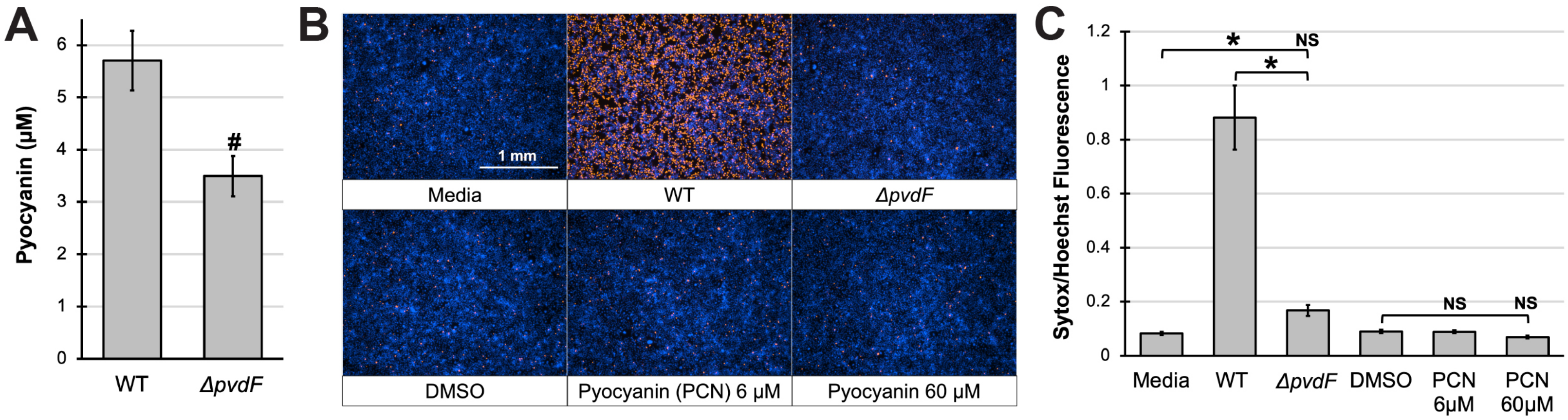

Fig. S4. Pyocyanin content in the conditioned medium is not sufficient for 16HBE cell death. (A) Concentration of pyocyanin in EMEM conditioned medium from WT PAO1 or PAO1*ΔpvdF*. **(B)** Fluorescent micrographs of 16HBE cells after 15 min exposure to conditioned medium from WT PAO1 or PAO1*ΔpvdF* or EMEM supplemented with purified pyocyanin. Cells were prelabeled with Hoechst 33342 nucleic acid stain (blue). **(C)** Quantification of Sytox Orange mean fluorescence intensity normalized to that of Hoechst 33342 in **(B)**. All error bars represent SEM from four biological replicates. * corresponds to $p < 0.01$, # corresponds to $p < 0.05$, and NS corresponds to $p > 0.05$ based on Student's *t*-test **(A)** or one-way ANOVA with Tukey's multiple comparisons test **(C)**.

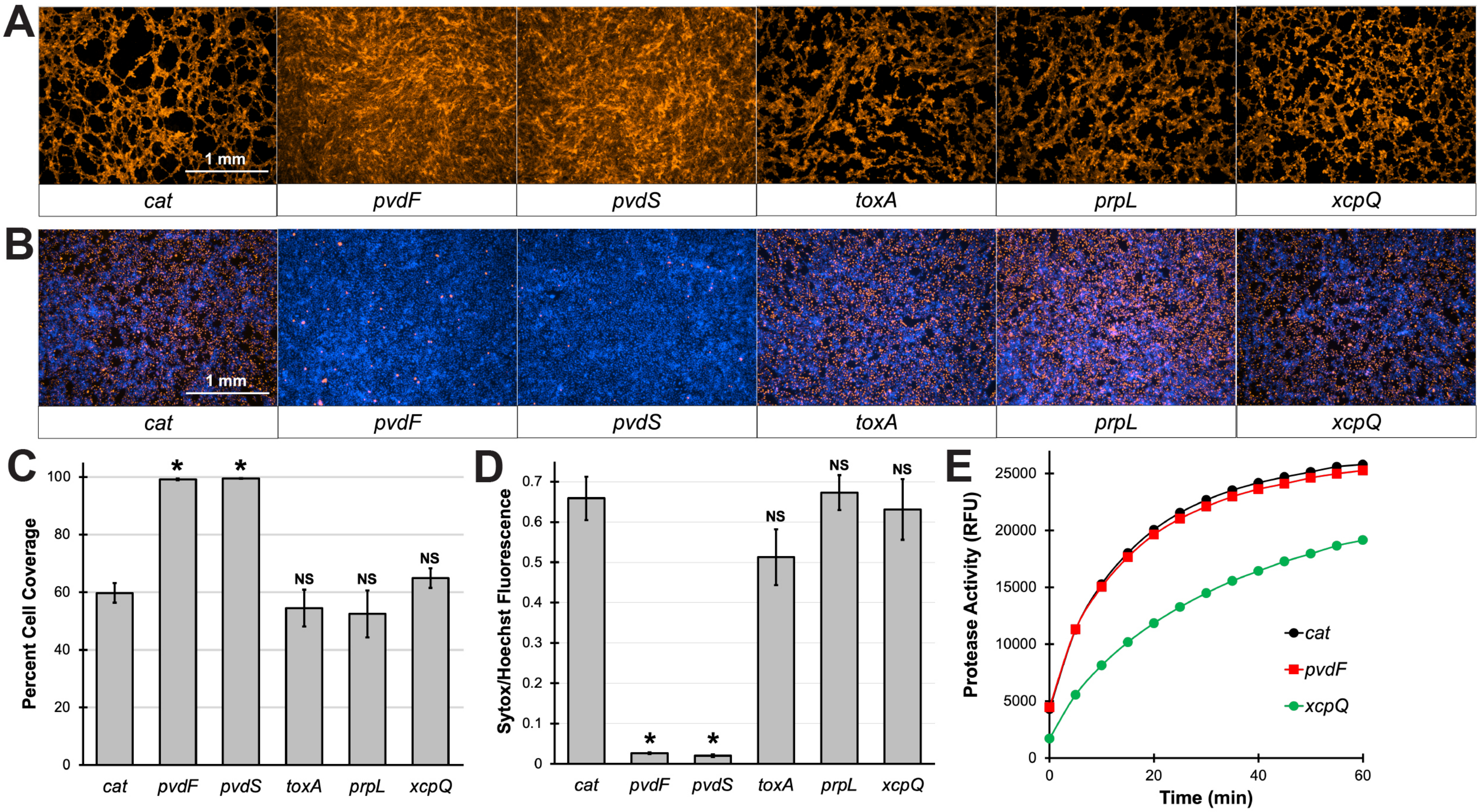

Fig. S5. Type II secretion system toxins do not contribute to lung epithelial damage. (A) Fluorescent micrographs of 16HBE cells after 30 min exposure to conditioned medium from MPAO1 transposon mutants. Cells were prelabeled with CellMask Orange plasma membrane stain. **(B)** Fluorescent micrographs of 16HBE cells after 15 min exposure to conditioned medium from MPAO1 transposon mutants in the presence of Sytox Orange nucleic acid stain (red). Cells were prelabeled with Hoechst 33342 nucleic acid stain (blue). **(C)** Quantification of percentage micrograph area covered by fluorescent cells in **(A)**. **(D)** Quantification of Sytox Orange mean fluorescence intensity normalized to that of Hoechst 33342 in **(B)**. **(E)** Protease activity in conditioned medium from MPAO1 transposon mutants. Proteolytic activity was measured by fluorescence release from cleavage of FITC-conjugated casein. All error bars represent SEM from three biological replicates. * corresponds to $p < 0.01$ and NS corresponds to $p > 0.05$ based on one-way ANOVA with Dunnett's multiple comparisons test.
