# Supplementary material for: In vitro lung epithelial cell model reveals novel roles for Pseudomonas aeruginosa siderophores
Source: Microbiol Spectr. 2024 Feb 5;12(3):e03693-23. doi: 10.1128/spectrum.03693-23 (PMC10913452; doi:10.1128/spectrum.03693-23)
Supplement: Figures S6-S10 — Supplemental figures S6-S10 with their legends. [file spectrum.03693-23-s0002.pdf]

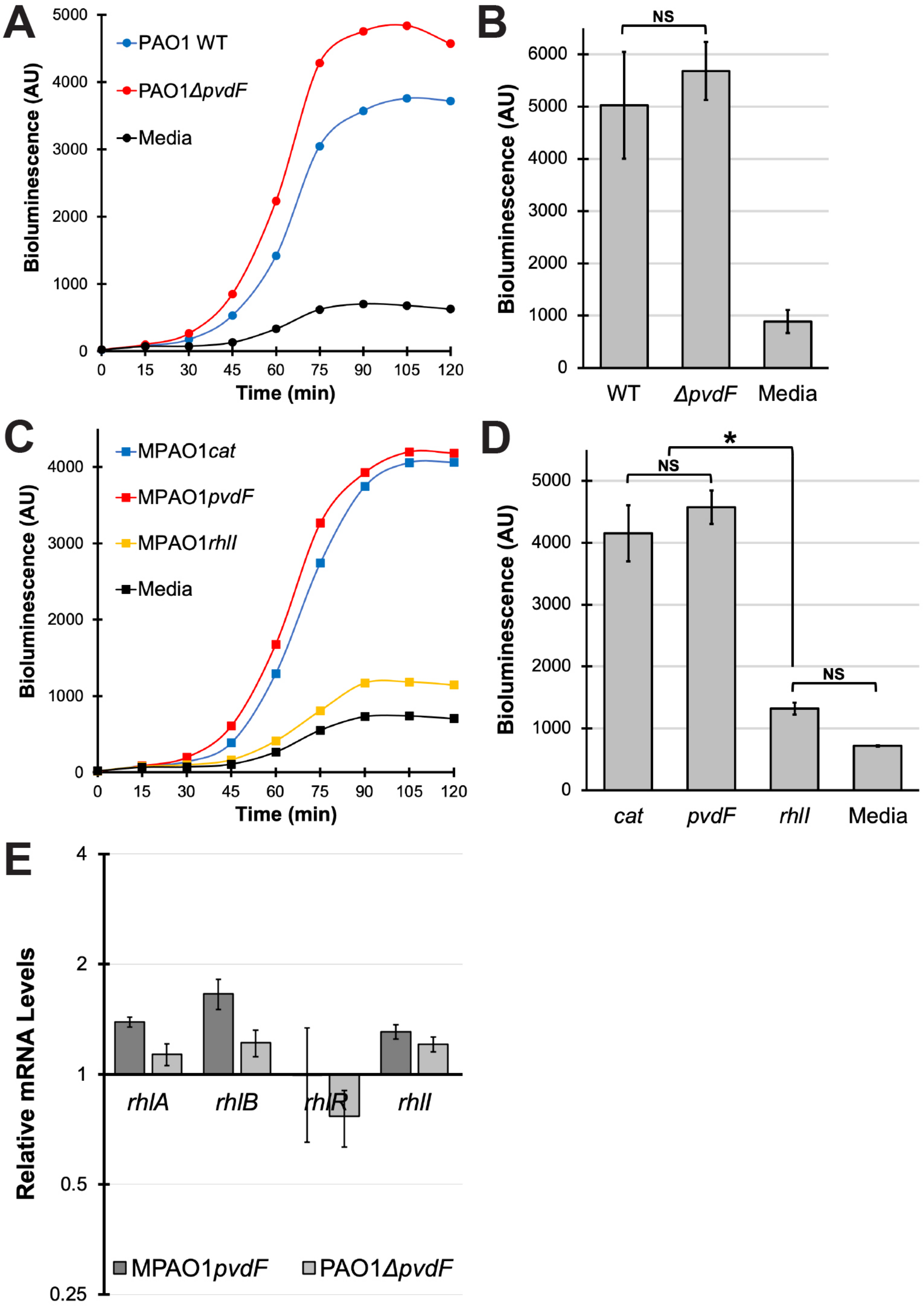

Fig. S6. Pyoverdine biosynthetic mutants do not exhibit impaired C4-HSL quorum-sensing. (A) Bioluminescence produced by *E. coli* JM109 pSB536 (N-butanoyl-L-homoserine lactone reporter strain) grown in media supplemented with conditioned medium from WT PAO1 or PAO1*ΔpvdF*. **(B)** Bioluminescence at the kinetics **(A)** endpoint. **(C)** Bioluminescence produced by the *E. coli* reporter strain grown in media supplemented with conditioned medium from MPAO1 transposon mutants. **(D)** Bioluminescence at the kinetics **(C)** endpoint. **(E)** Expression of rhamnolipid biosynthetic (*rhlA*, *rhlB*) and regulatory (*rhlR*, *rhlI*) genes in MPAO1*pvdF* or PAO1*ΔpvdF* normalized to that of WT PAO1. All error bars represent SEM from at least three biological replicates. * corresponds to $p < 0.01$ and NS corresponds to $p > 0.05$ based on one-way ANOVA with Tukey's multiple comparisons test.

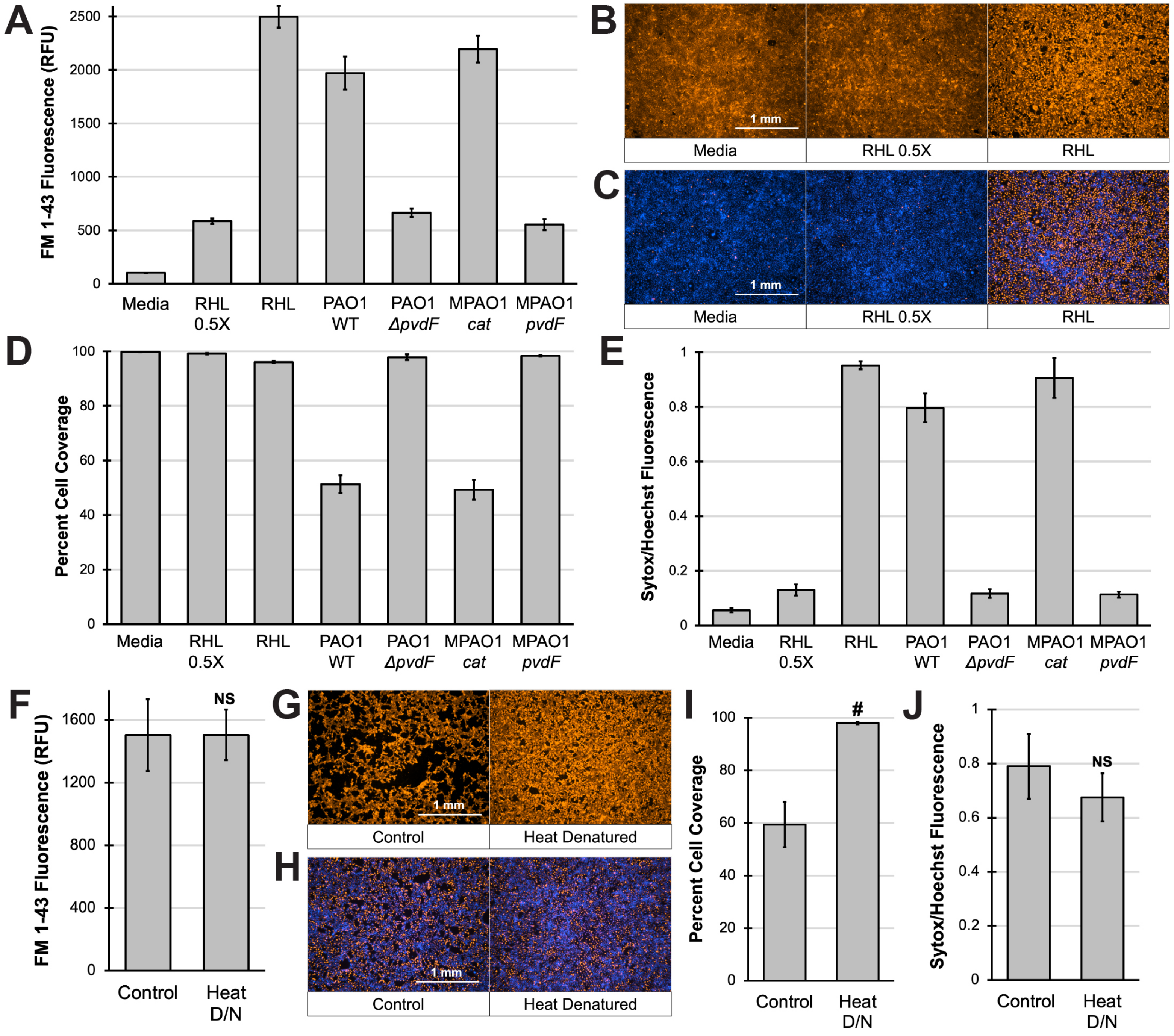

Fig. S7. Purified rhamnolipids are sufficient for 16HBE cell death but not for epithelial monolayer damage. (A) Quantification of rhamnolipids in purified samples (RHL) or *P. aeruginosa* conditioned medium from WT PAO1, PAO1*ΔpvdF*, MPAO1*cat*, or MPAO1*pvdF*. **(B)** Fluorescent micrographs of 16HBE cells after 30 min exposure to purified rhamnolipids in EMEM. Cells were prelabeled with CellMask Orange plasma membrane stain. **(C)** Fluorescent micrographs of 16HBE cells after 15 min exposure to purified rhamnolipids in EMEM in the presence of Sytox Orange nucleic acid stain (red). Cells were prelabeled with Hoechst 33342 nucleic acid stain (blue). **(D)** Quantification of percentage micrograph area covered by fluorescent cells in **(B)**. **(E)** Quantification of Sytox Orange mean fluorescence intensity normalized to that of Hoechst 33342 in **(C)**. **(F)** Quantification of rhamnolipids in conditioned medium from WT PAO1 or conditioned medium after heat denaturation (85 °C for 1 h). **(G)** Fluorescent micrographs of 16HBE cells after 30 min exposure to conditioned medium from WT PAO1 or conditioned medium after heat denaturation. Cells were prelabeled with CellMask Orange plasma membrane stain. **(H)** Fluorescent micrographs of 16HBE cells after 15 min exposure to conditioned medium from WT PAO1 or conditioned medium after heat denaturation in the presence of Sytox Orange nucleic acid stain (red). Cells were prelabeled with Hoechst 33342 nucleic acid stain (blue). **(I)** Quantification of percentage micrograph area covered by fluorescent cells in **(G)**. **(J)** Quantification of Sytox Orange mean fluorescence intensity normalized to that of Hoechst 33342 in **(H)**. All error bars represent SEM from three biological replicates. # corresponds to $p < 0.05$ and NS corresponds to $p > 0.05$ based on Student's t-test.

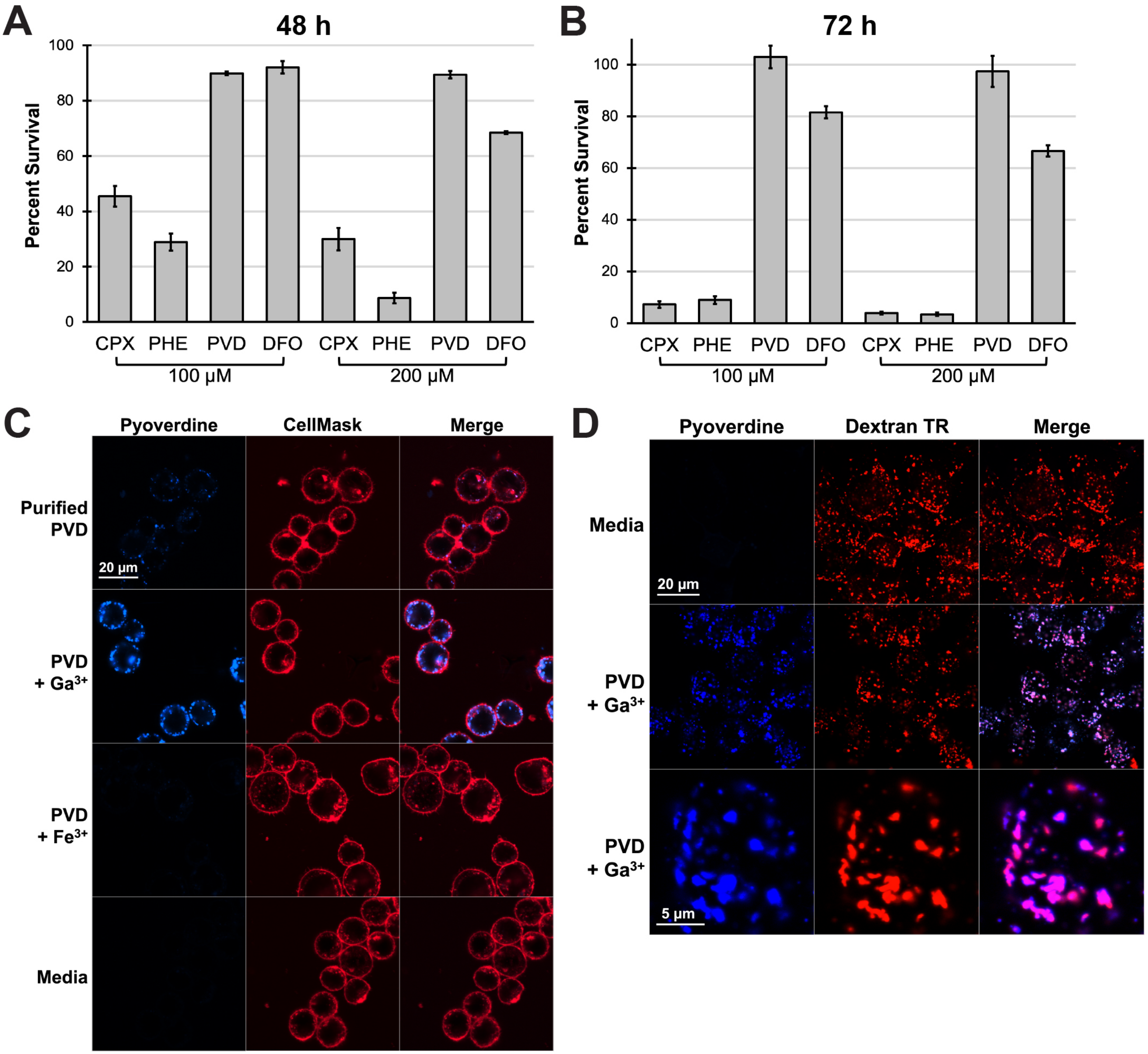

Fig. S8. Pyoverdine accumulates in early endosomes of lung epithelial cells. (A, B) 16HBE cell viability after 48 **(A)** or 72 h **(B)** treatment with ciclopirox olamine, 1, 10-phenanthroline, pyoverdine, or deferoxamine in serum-free EMEM. **(C)** Confocal micrographs of 16HBE cells exposed to 100 μM purified pyoverdine, pyoverdine with excess $Ga(NO_3)_3$, pyoverdine with excess $FeCl_3$, or media control for 24 h. Cells were labeled with CellMask Deep Red plasma membrane stain and trypsinated prior to imaging. **(D)** Confocal micrographs of 16HBE cells treated with 100 μM pyoverdine-gallium and dextran-Texas Red (10,000 MW). Bottom row shows an enlarged micrograph of one representative cell. All error bars represent SEM from three biological replicates.

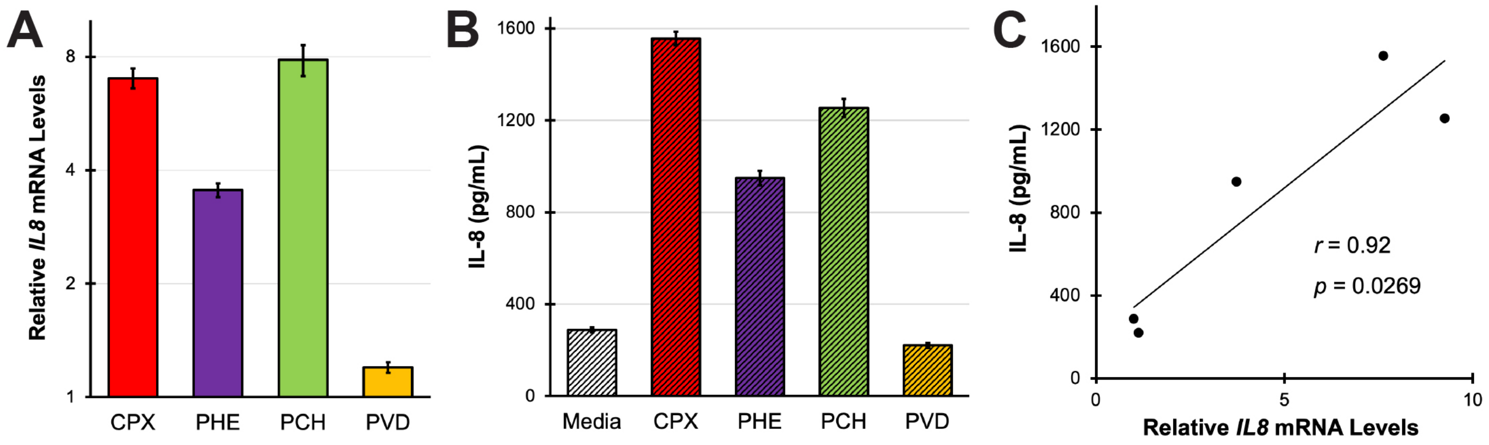

Fig. S9. Small molecule iron chelators promote IL-8 production in 16HBE cells. (A) *IL8* mRNA levels in 16HBE cells treated with iron chelators (ciclopirox olamine – CPX, 1,10-phenanthroline – PHE, pyochelin – PCH, pyoverdine – PVD) for 30 h compared to that of media control. mRNA levels were measured by qRT-PCR. **(B)** IL-8 protein concentration in the supernatants of 16HBE cells treated with iron chelators for 48 h. IL-8 was quantified by ELISA. **(C)** Correlation between *IL8* gene expression and IL-8 protein production. All error bars represent SEM from three biological replicates.

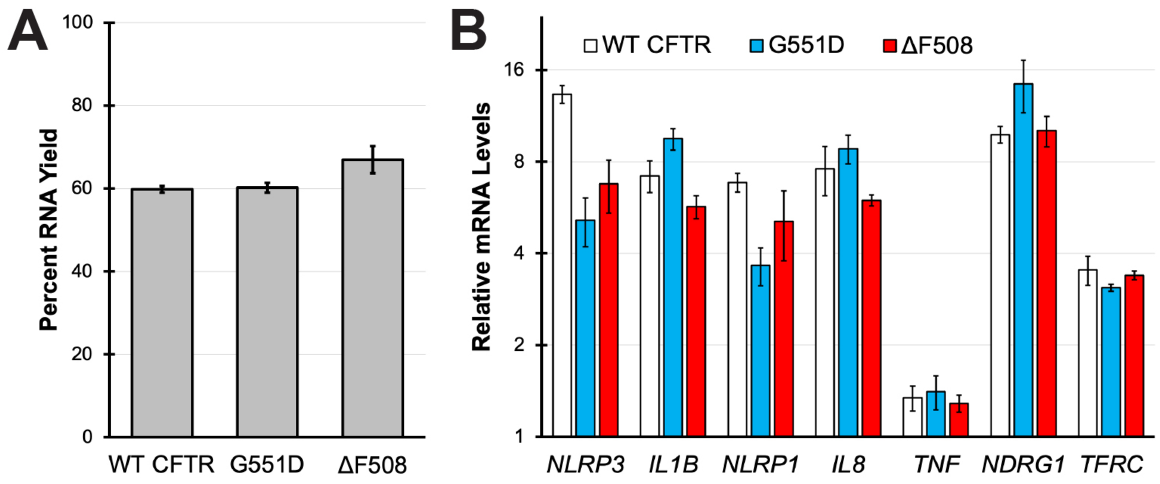

Fig. S10. Deferoxamine promotes the expression of proinflammatory genes in 16HBE CFTR mutants. (A) Total RNA yield in WT 16HBE cells and 16HBE cells carrying mutations (G551D or ΔF508) in the cystic fibrosis transmembrane conductance regulator (CFTR) after 48 h treatment with 100 μM deferoxamine in serum-free EMEM. **(B)** Expression of proinflammatory genes (*NLRP3, IL1B, NLRP1, IL8, TNF*) or iron-regulated genes (*NDRG1*, *TFRC*) in 16HBE cells treated with deferoxamine compared to that of media control. mRNA levels were measured by qRT-PCR. All error bars represent SEM from three biological replicates.
