# Supplementary material for: In vitro lung epithelial cell model reveals novel roles for Pseudomonas aeruginosa siderophores
Source: Microbiol Spectr. 2024 Feb 5;12(3):e03693-23. doi: 10.1128/spectrum.03693-23 (PMC10913452; doi:10.1128/spectrum.03693-23)
Supplement: Table S1 [file spectrum.03693-23-s0003.pdf]

Primer Name	Sequence (5' - 3')	Description
ARB1D	GGCCAGGCCTGCAGATGATGNNNNNNNNNNGTAT	MPAO1 Tn Verification Arbitrary PCR Round 1
PhoA Tn_1	GTTAACCATAACTTCGTATAATG	MPAO1 Tn Verification Arbitrary PCR Round 1 (ISphoA/hah)
LacZ Tn_1	GTAAACTGGATGGCTTTCTTGCC	MPAO1 Tn Verification Arbitrary PCR Round 1 (ISlacZ/hah)
ARB2A	GGCCAGGCCTGCAGATGATG	MPAO1 Tn Verification Arbitrary PCR Round 2
PhoA Tn_2	AATTGGATAACTTCGTATAATGTATGC	MPAO1 Tn Verification Arbitrary PCR Round 2 (ISphoA/hah)
LacZ Tn_2	AAGGATCTGATGGCGCAGGGGATCCCC	MPAO1 Tn Verification Arbitrary PCR Round 2 (ISlacZ/hah)
MPAO1_Seq	GTTATTAATTAAGCATCACC	MPAO1 Tn Verification Sanger Sequencing Primer
gyrB_F	CCTGCTGTTGACCTTCTTCT	*P. aeruginosa gyrB* qRT-PCR - Forward
gyrB_R	CTGGTCGTCCTTGATGTACTG	*P. aeruginosa gyrB* qRT-PCR - Reverse
rhlA_F	CGAGACCGTCGGCAAATAC	*P. aeruginosa rhlA* qRT-PCR - Forward
rhlA_R	GCACCTGGTCGATGTGAAA	*P. aeruginosa rhlA* qRT-PCR - Reverse
rhlB_F	CTCACGAGAAGTACGGGATTC	*P. aeruginosa rhlB* qRT-PCR - Forward
rhlB_R	CTCGGGCACGTTGAACT	*P. aeruginosa rhlB* qRT-PCR - Reverse
rhlR_F	ATTTGCTCAGCGTGCTTTC	*P. aeruginosa rhlR* qRT-PCR - Forward
rhlR_R	GGGTCAGCAACTCGATCAT	*P. aeruginosa rhlR* qRT-PCR - Reverse
rhlI_F	TACCTGTGCAGCGAAACC	*P. aeruginosa rhlI* qRT-PCR - Forward
rhlI_R	GCAGGCTGGACCAGAATATC	*P. aeruginosa rhlI* qRT-PCR - Reverse
ACTB_F	CACCATTGGCAATGAGCGGTTC	16HBE *ACTB* qRT-PCR - Forward
ACTB_R	AGGTCTTTGCGGATGTCCACGT	16HBE *ACTB* qRT-PCR - Reverse
NLRP3_F	GGACTGAAGCACCTGTTGTGCA	16HBE *NLRP3* qRT-PCR - Forward
NLRP3_R	TCCTGAGTCTCCCAAGGCATTC	16HBE *NLRP3* qRT-PCR - Reverse
IL1B_F	CCACAGACCTTCCAGGAGAATG	16HBE *IL1B* qRT-PCR - Forward
IL1B_R	GTGCAGTTCAGTGATCGTACAGG	16HBE *IL1B* qRT-PCR - Reverse
NRLP1_F	ATTGAGGGCAGGCAGCACAGAT	16HBE *NLRP1* qRT-PCR - Forward
NRLP1_R	CTCCTTCAGGTTTCTGGTGACC	16HBE *NLRP1* qRT-PCR - Reverse
IL8_F	GAGAGTGATTGAGAGTGGACCAC	16HBE *IL8* qRT-PCR - Forward
IL8_R	CACAACCCTCTGCACCCAGTTT	16HBE *IL8* qRT-PCR - Reverse
TNF_F	CTCTTCTGCCTGCTGCACTTTG	16HBE *TNF* qRT-PCR - Forward
TNF_R	ATGGGCTACAGGCTTGTCACTC	16HBE *TNF* qRT-PCR - Reverse
NDRG1_F	ATCACCCAGCACTTTGCCGTCT	16HBE *NDRG1* qRT-PCR - Forward
NDRG1_R	GACTCCAGGAAGCATTTCAGCC	16HBE *NDRG1* qRT-PCR - Reverse
TFRC_F	ATCGGTTGGTGCCACTGAATGG	16HBE *TFRC* qRT-PCR - Forward
TFRC_R	ACAACAGTGGGCTGGCAGAAAC	16HBE *TFRC* qRT-PCR - Reverse

Table S1. List of primers used in this study.
